# Clinical outcomes and prognostic factors to predict treatment response in high risk neuroblastoma patients receiving topotecan and cyclophosphamide containing induction regimen: a prospective multicenter study

**DOI:** 10.1186/s12885-019-6186-z

**Published:** 2019-10-16

**Authors:** Piya Rujkijyanont, Apichat Photia, Chanchai Traivaree, Chalinee Monsereenusorn, Usanarat Anurathapan, Panya Seksarn, Darintr Sosothikul, Piti Techavichit, Kleebsabai Sanpakit, Kamon Phuakpet, Surapon Wiangnon, Thirachit Chotsampancharoen, Su-on Chainansamit, Somjai Kanjanapongkul, Arunotai Meekaewkunchorn, Suradej Hongeng

**Affiliations:** 1 Division of Hematology-Oncology, Department of Pediatrics, Phramongkutklao Hospital and College of Medicine, 315 Ratchawithi Road, Ratchathewi, Bangkok, 10400 Thailand; 2 Division of Hematology-Oncology, Department of Pediatrics, Faculty of Medicine, Ramathibodi Hospital, Mahidol University, Bangkok, Thailand; 3 Division of Hematology-Oncology, Department of Pediatrics, Faculty of Medicine, Chulalongkorn University, Bangkok, Thailand; 4 Division of Hematology-Oncology, Department of Pediatrics, Faculty of Medicine, Siriraj Hospital, Mahidol University, Bangkok, Thailand; 5 Faculty of Medicine, Mahasarakham University, Mahasarakham, Thailand; 6 Division of Hematology-Oncology, Department of Pediatrics, Faculty of Medicine, Prince of Songkla University, Hat Yai, Thailand; 7 Department of Pediatrics, Khon Kaen Hospital, Khon Kaen, Thailand; 8 Division of Hematology-Oncology, Queen Sirikit National Institute of Child Health, Bangkok, Thailand

**Keywords:** High risked neuroblastoma, Topotecan, Induction therapy, Treatment response, Prognostic factor, Treatment-related toxicity

## Abstract

**Background:**

Neuroblastoma is the most common extra-cranial solid tumor among children. Despite intensive treatment, patients with advanced disease mostly experience dismal outcomes. Here, we proposed the use of topotecan and cyclophosphamide containing induction regimen as an upfront therapy to high risk neuroblastoma patients.

**Methods:**

Patients with high risk neuroblastoma undergoing ThaiPOG high risk neuroblastoma protocol from 2016 to 2017 were studied. All patients received 6 cycles of induction regimen consisting of 2 cycles topotecan (1.2 mg/m^2^/day) and cyclophosphamide (400 mg/m^2^/day) for 5 days followed by cisplatin (50 mg/m^2^/day) for 4 days combined with etoposide (200 mg/m^2^/day) for 3 days on the third and fifth cycles and cyclophosphamide (2100 mg/m^2^/day) for 2 days combined with doxorubicin (25 mg/m^2^/day) and vincristine (0.67 mg/m^2^/day) for 3 days on the fourth and sixth cycles. Treatment response after the 5th cycle before surgery and treatment-related toxicities after each topotecan containing induction cycle were evaluated. Relevant prognostic factors were analyzed to measure the treatment response among those patients.

**Results:**

In all, 107 high risk neuroblastoma patients were enrolled in the study. After the 5th cycle of induction regimen, the patients achieved complete response (*N* = 2), very good partial response (*N* = 40), partial response (*N* = 46) and mixed response (*N* = 19). None of the patients experienced stable disease or disease progression. The most significant prognostic factor was type of healthcare system. The most common adverse effect was febrile neutropenia followed by mucositis, diarrhea and elevated renal function.

**Conclusion:**

The topotecan and cyclophosphamide containing induction regimen effectively provides favorable treatment response. The regimen is well tolerated with minimal toxicity among patients with high risk neuroblastoma in Thailand.

## Background

Neuroblastoma is a cancer of autonomic nervous system and the most common cancer during infancy. Clinical presentations among patients with neuroblastoma vary depending on tumor location although most patients present with suprarenal mass [1]. The diagnosis of neuroblastoma can be made by either tissue pathology or evidence of bone marrow involvement with increased urine catecholamine metabolites. The predictive and prognostic factors influencing a patient’s response to treatment and overall outcomes include age, disease stage at diagnosis, tumor histology, DNA ploidy, MYCN (n-myc) status and chromosome changes [2]. Due to the high heterogeneity of neuroblastoma, treatment strategies are decided based on risk stratification according to the International Neuroblastoma Risk Group Staging System (INRGSS) ranging from close observation without intervention among low risk patients to a combination of chemotherapy, surgery and radiation with or without hematopoietic stem cell transplantation among high risk patients [3–5]. In addition, treatment outcomes in each individual are also diverse ranging from excellent outcomes with spontaneous tumor regression among low risk patients to extremely unfavorable prognosis with a survival rate of less than 20–30% despite a combination of intensive treatment among high risk patients [1, 5–7].

Among patients with high risk neuroblastoma, additional treatment options such as anti-GD2 (hu14.18K322A) immunotherapy, iodine 123 (123I) metaiodobenzylguanidine (MIBG) treatment and cellular therapy such as adapting chimeric antigen receptor (CAR) T-cell and natural killer (NK) cell therapy have been studied and incorporated to standard treatment with very promising results [8–10]. However, the unavailability of those additional treatments in many institutions with limited resources especially in developing countries becomes a challenging dilemma for healthcare providers to improve clinical outcomes of those patients with high risk neuroblastoma. Evidence has shown that the level of response was a significant predictor of the patient’s long-term outcomes even in the non-hematopoietic stem cell transplantation setting. The even-free survival for patients receiving chemotherapy without hematopoietic stem cell transplantation who achieved complete response (CR) or very good partial response (VGPR) was significantly superior to even-free survival of those who achieved partial response or lower [11]. Therefore, in resource-limited settings, developing a new chemotherapy regimen by introducing highly potent cytotoxic and tumor-specific agents might be the major key element to improve outcomes among those high risk patients.

Topotecan is a water-soluble semi-synthetic derivative of camptothecin and acts as a topoisomerase I inhibitor. This drug exerts its cytotoxic effects during the S-phase of DNA synthesis by binding to the topoisomerase I – DNA complex and inhibiting relegation of this single-strand break leading to replication arrest and apoptosis. In addition to the approval indication in treating ovarian cancer, cervical cancer and small cell lung cancer [12–14], topotecan has been studied among children with recurrent and refractory malignant solid tumors with promising results in which complete response plus partial response were successfully obtained regarding rhabdomyosarcoma, Ewing’s sarcoma and neuroblastoma patients [15, 16]. The common side effects of topotecan include myelosuppression, diarrhea, constipation, nausea, vomiting and stomatitis. Among patients diagnosed with recurrent and refractory high risk neuroblastoma, a combination of topotecan and cyclophosphamide or topotecan and etoposide was found to be effective and tolerable [17–19]. In addition, a combination of topotecan and cyclophosphamide was found to be superior to topotecan monotherapy in improving progression free survival among recurrent and patients with refractory high risk neuroblastoma [20]. The use of topotecan and cyclophosphamide as an upfront induction regimen in newly diagnosed high risk neuroblastoma patients was firstly introduced over a decade ago [21]. According to the published data from the Children’s Oncology Group, the induction regimen was found to be well-tolerated with reversible toxicities including hematologic toxicity and febrile neutropenia. At the end of induction, 26 of 31 patients achieved tumor response with 1 patient experienced progressive disease [22]. A higher dose of topotecan and cyclophosphamide containing induction regimen in de novo high risk neuroblastoma patients was also studied; however, the induction responses were comparable to those from the Children’s Oncology Group in which 29 of 34 patients achieved tumor response (12 patients achieved a partial remission and 17 patients achieved complete remission/very good partial remission) [23]. In this study, we conducted a nationwide multicenter clinical trial and incorporated a combination of topotecan and cyclophosphamide as upfront induction chemotherapy for patients with de novo high risk neuroblastoma and reported clinical outcomes including post induction response, treatment-related toxicities as well as classified relevant prognostic factors to measure patients’ induction responses.

## Methods

### Patient selection

One hundred and seven pediatric patients with newly diagnosed high risk neuroblastoma in Thailand from January 2016 to December 2017 were enrolled in this study. Written informed consent and assent forms to participate in the study were obtained from all participating subjects including children, their parents or legal guardians before enrolling in this study. This clinical study was approved by the Institutional Review Board according to the ethical principles of the Declaration of Helsinki (1975) including its revision. The study’s inclusion criteria included patients less than 18 years of age who had a new diagnosis of high risk neuroblastoma stratified as per the International Neuroblastoma Risk Group Staging System (INRGSS). Patients contra-indicated for topotecan or receiving a diagnosis of refractory or relapsed neuroblastoma were excluded from the study.

### Topotecan and cyclophosphamide containing induction regimen

All patients with de novo high risk neuroblastoma enrolled in this study underwent treatment according to the Thai Pediatric Oncology Group (ThaiPOG) protocol for high risk neuroblastoma (ThaiPOG-NB-13HR) in which the induction regimen was referred to that used in the Children’s Oncology Group study (ANBL0532) [24]. Induction regimen included six cycles of multiagent chemotherapy given every 21 days or thereafter when absolute neutrophil count (ANC) was ≥1000 cells/mm^3^ and platelet count was ≥75,000 cells/mm^3^. Subcutaneous granulocyte colony-stimulating factor 5 μg/kg/dose was given daily starting 24 to 48 h after completing chemotherapy on each cycle and continued until ANC recovery of greater than 1000/mm^3^ post nadir. All chemotherapy doses were calculated according to body-surface area (m^2^) for patients weighing > 12 kg or based on weight (kg) for those weighing ≤12 kg. The first two induction cycles were topotecan-based consisting of intravenous topotecan 1.2 mg/m^2^ (0.04 mg/kg) combined with cyclophosphamide 400 mg/m^2^ (13.3 mg/kg) and given daily for 5 days. The third and fifth induction cycles comprised intravenous cisplatin 50 mg/m^2^ (1.66 mg/kg) given daily for 4 days combined with etoposide 200 mg/m^2^ (6.67 mg/kg) given daily for 3 days. The fourth and sixth induction cycles included intravenous cyclophosphamide 2100 mg/m^2^ (70 mg/kg) given daily for 2 days combined with doxorubicin 25 mg/m^2^ (0.83 mg/kg) given daily for 3 days, and vincristine 0.67 mg/m^2^ (0.022 mg/kg among patients ≥12 months of age or 0.017 mg/kg among patients < 12 months of age regardless of body weight) given daily for 3 days.

### Induction response criteria

All high risk neuroblastoma patients participated in this study underwent initial evaluation before initiating treatment including imaging studies of the primary tumor as well as metastatic sites, bone scan and bone marrow examination according to INRGSS. Tumor histology and differentiation, MYCN (n-myc) status (amplified versus non-amplified) and Shimada histology were also performed as a part of initial disease staging. However, MYCN status might not have been performed in all patients who had clear evidence of distant metastasis because those patients would be classified as a high risk neuroblastoma group regardless of MYCN status. Follow-up evaluation including CT scan, bone marrow and bone scintigraphy was then repeated at the end of the fifth cycle of chemotherapy to evaluate treatment response before surgical resection of the tumor. In addition, urine vanillylmandelic acid (VMA) and serum neuron-specific enolase (NSE) were obtained at the time of initial diagnosis and serially repeated with each cycle of induction chemotherapy. Treatment response was determined by treating physicians, and imaging studies were evaluated by radiologists from each institution. Due to the limited access to MIBG and PET/CT scans in some patients/institutions, bone scintigraphy was mainly used in this study to evaluate bone involvement and the original international neuroblastoma response criteria (INRC) of Brodeur et al., 1993 [25] was used to assess treatment response instead of the revised version of INRC of Park et al., 2017 [26] which essentially requires information from MIBG and PET/CT scans. According to the original INRC, complete response (CR) was characterized as no evidence of primary tumor and metastatic lesions (chest, abdomen, liver, lymph nodes, bone marrow, central nervous system, etc.). A very good partial response (VGPR) was characterized as decreased primary tumor’s size by 90 to 99% with no metastatic lesions (except bone), no new bone lesions and improved all pre-existing lesions. A partial response (PR) was characterized as decreased primary tumor’s size > 50% with no new metastatic lesions and 50 to 90% reduction in measurable sites, 0 to 1 bone marrow samples with tumor and bone lesions similar to VGPR. A mixed response (MR) was characterized as no new lesions at primary and metastatic sites with > 50% reduction of any measurable lesions (primary or metastases) with < 50% reduction in any others or < 25% increase in any existing lesions. No response (NR) was characterized as no new lesions at primary and metastatic sites with < 50% reduction and < 25% increase in any existing lesions. Finally, progressive disease (PD) was characterized as any new lesions or increase of any measurable lesions by 25% or previously negative marrow positives for tumor.

### Measurement of treatment-related toxicities

Treatment-related adverse effects such as febrile neutropenia, bone marrow suppression, mucositis, typhlitis, diarrhea and hepatorenal toxicity were evaluated among enrolled patients during and after each topotecan-containing chemotherapy cycle (first and second cycles of induction treatment). Bone marrow suppression was characterized as an absolute neutrophil count of < 500 cell/mm^3^ within 3 days and/or platelet count of < 75,000 cells/mm^3^ within 1 week after the cycle of chemotherapy. Febrile neutropenia was characterized as an oral temperature of > 38.5 °C or two consecutive readings of > 38.0 °C for 2 h with an absolute neutrophil count of < 500 cells/mm^3^, or expected to fall below 500 cells/mm^3^. Renal toxicity was characterized as increased level of serum creatinine >2SD of the upper limit of normal in the reference population at the same age. Liver toxicity was characterized as increased level of serum aspartate aminotransferase (AST) and/or serum alanine aminotransferase (ALT) >2SD of the upper limit of normal in the reference population at the same age. In addition, duration of hospitalization and re-admission after chemotherapy were collected among all patients during and after each topotecan-containing chemotherapy cycle.

### Statistical analysis

Baseline values of selected variables were analyzed and presented as mean with standard deviation (SD) or median (range) for continuous variables and were calculated using frequency and percentage for categorical variables. Univariate and multivariate analyses were performed using logistic regression to analyze the prognostic factors to predict treatment response after topotecan and cyclophosphamide containing induction regimen. Treatment-related toxicities after each cycle of topotecan and cyclophosphamide containing induction therapy were calculated using frequency and percentage for categorical variables. Statistical Package for the Social Science (SPSS), Version 23 Software (IBM, NY, USA) was used and a *p*-value < 0.05 was considered significant.

## Results

### Patient characteristics

Patient’ characteristics including age, sex, primary tumor site, metastatic site, serum NSE and urine VMA levels, location and types of health care system (university-based versus community-based health care system) are summarized in Table 1. The patients’ ages in this study resembled a typical age range in neuroblastoma. Males were more predominant than females at a ratio of 1.3:1. The most common primary tumor site was the adrenal gland followed by thoracic and abdominal origins, in rank order. Among 107 high risk neuroblastoma patients enrolled in this study, 106 were stage M or metastatic high risk patients and 1 was stage L2 non-metastatic high risk patients with MYCN amplification. The most common initial metastatic sites at diagnosis were the bone marrow, bone and lymph node in rank order. Surgical resection of the tumor at diagnosis was performed in 31 of 107 high risk neuroblastoma patients and 15 of 40 patients who achieved VGPR. Three fourths of the patients were treated at a university-based health care system, and the remaining patients were treated at a community-based health care system. Patients’ locations were equally distributed among different parts of Thailand. Pathologic findings of tumor including tumor histology, tumor differentiation, MYCN status (amplified versus non-amplified) and Shimada histology are summarized in Table 2. The most common pathological finding of the tumor was poorly differentiated, unfavorable neuroblastoma. Since the neuroblastoma patients with metastatic disease is stratified in high risk group regardless of MYCN status from the tumor, MYCN test was not performed in those patients. MYCN status was evaluated among one half of the patients and one third of those specimens were MYCN-amplified.

**Table 1:** Patient characteristics

	*Patients (n = 107)*	*Numbers (%)*
*Age (years)*	Mean ± SD	6.0 ± 3.1
	Median (range)	5.3 (1.3–17.4)
*Gender*	Male	62 (57.9)
	Female	45 (42.1)
*Region*	Central	45 (42.1)
	Northeast	41 (38.3)
	South	21 (19.6)
*Health care system*	University-based	81 (75.7)
	Community-based	26 (24.3)
*Primary tumor site*	Adrenal	88 (82.2)
	Abdominal	4 (3.7)
	Thoracic	7 (6.5)
	Others	8 (7.5)
*Metastatic site at diagnosis*	Bone marrow	69 (64.5)
	Bone	66 (61.7)
	Lymph node	41 (38.3)
	Liver	14 (13.1)
	Brain	3 (2.8)
	Others	7 (6.5)
*Serum NSE (n = 82)*	Mean ± SD	455.4 ± 412.9
	Median (range)	370.0 (0–2180.0)
*Urine VMA (n = 86)*	Mean ± SD	38.2 ± 70.1
	Median (range)	18.9 (0–494.0)
*INRG Stage*	Stage M	106 (99.0)
	Stage L2 (with MYCN amplification)	1 (1.0)

*Notes: Data are presented as mean ± SD and median (range) for continuous variables and number (%) for categorical variables*

Abbreviations: *NSE* serum neuron-specific enolase (ng/mL); *VMA* urine vanillylmandelic acid (mg/day); *Stage M* distant metastatic disease (except stage MS); *Stage L2* locoregional tumor with presence of one or more image-defined risk factors

**Table 2:** Tumor characteristics

	*Data*	*Numbers (%)*
*Tumor histology* *(n = 107)*	Neuroblastoma	99 (92.5)
	Ganglioneuroblastoma, intermixed	8 (7.5)
*Tumor differentiation* *(n = 73)*	Undifferentiated	30 (41.2)
	Poorly differentiated	35 (47.9)
	Differentiated	8 (10.9)
*MYCN amplification* *(n = 51)*	MYCN non-amplified	39 (76.4)
	MYCN amplified	12 (23.5)
*Shimada histology* *(n = 32)*	Unfavorable	23 (71.9)
	Favorable	9 (28.1)

***Notes:*** *Data are presented as number (%) for categorical variables*

### Analysis of overall induction response

All high risk neuroblastoma patients underwent initial evaluation at the time of diagnosis before initiating treatment. Patients were re-evaluated and CT scan, bone marrow and bone scintigraphy were performed at the end of fifth cycle of induction treatment to determine treatment response and disease status before surgical resection of the tumor. Imaging studies were also obtained and compared with the initial studies. Interestingly, all 107 patients responded to induction treatment in which 2 and 40 of those successfully obtained CR and VGPR while the remaining patients achieved PR and MR. In addition, all 40 patients who achieved VGPR had bone involvement at initial diagnosis, and 20 of those experienced complete resolution of bone lesions after the 5th induction cycle whereas 18 and 2 of those had decreased and stable bone lesions respectively. None of the patients who achieved VGPR had an evidence of progression on bone scintigraphy. Moreover, none of the high risk patients enrolled in the study had NR and PD **(**Table 3**)**.

**Table 3:** Induction treatment response (*n* = 107)

*Treatment response*	*N (%)*
Complete response (CR)	2 (1.9)
Very good partial response (VGPR)	40 (37.4)
Partial response (PR)	46 (43)
Mixed response (MR)	19 (17.8)
No response (NR)	–
Progressive disease (PD)	–

***Notes:*** *Data are presented as number (%) for categorical variables*

### Prognostic factors to predict induction response

**Fig. 1:**
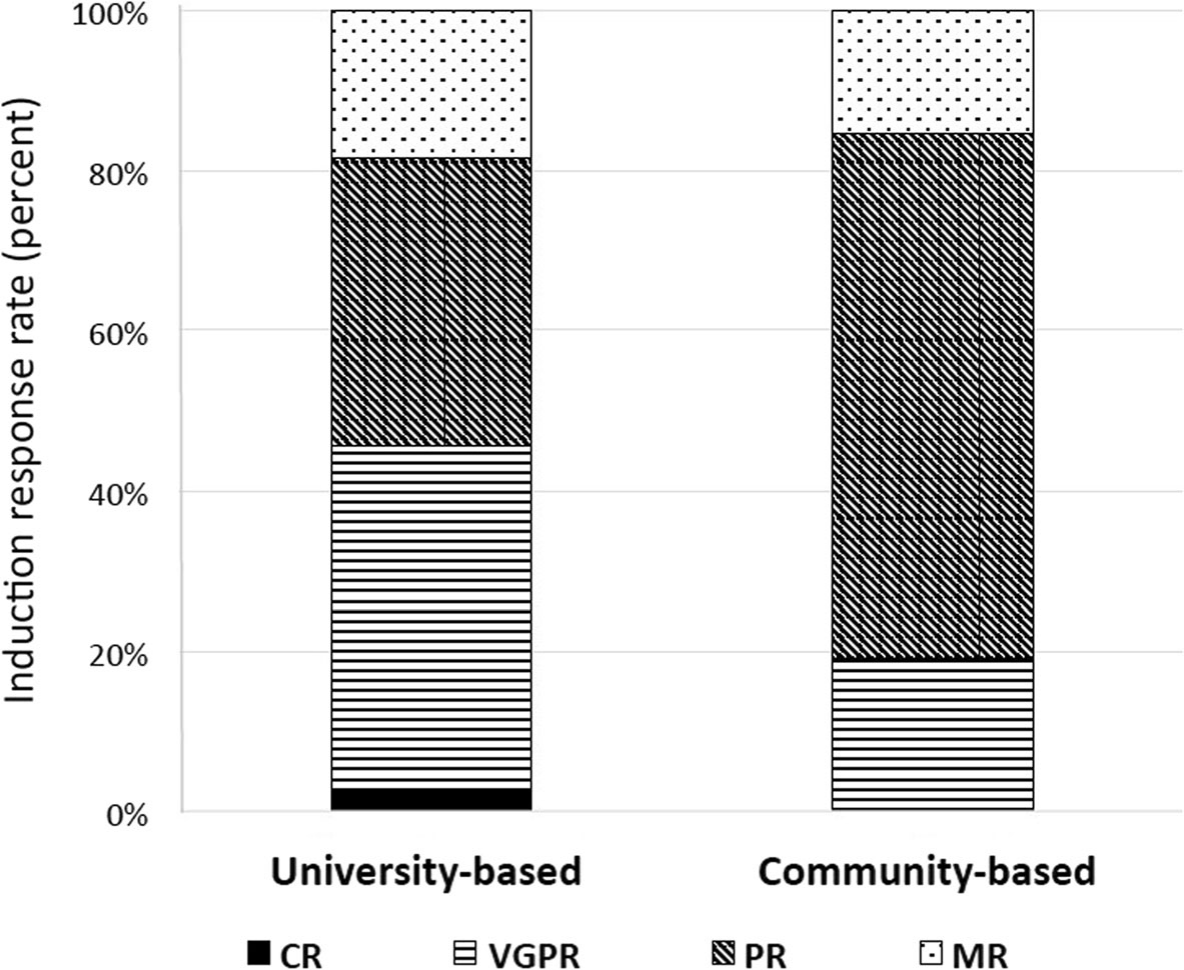
Induction response based on health care systems (university- versus community-based health care system). ***Notes:*** *Graphs are shown as number (%)*. ***Abbreviations:*** *CR, complete response; VGPR, very good partial response; PR, partial response; MR, mixed response*

Associated factors from all patients with high risk neuroblastoma including patient’s age, sex, grade of tumor differentiation, MYCN status, Shimada histology, serum NSE, urine VMA and type of health care system were analyzed to determine predictive factors of treatment response after topotecan and cyclophosphamide containing induction regimen. However, we found no association between those associated factors and induction response, except for type of health care system **(**Table 4**)**. Interestingly, high risk patients, treated at a university-based health care system, successfully achieved a significantly treatment response (≥90% tumor reduction in almost half of the patients) than those treated at a community-based health care system (≥90% tumor reduction in one fifth of the patients) with a *p*-value of 0.021. Induction responses based on type of health care system (university-based versus community-based health care system) are shown in Fig. 1. Multivariate analysis was performed using multiple logistic regression adjusted for age, sex, grade of tumor differentiation, MYCN status, Shimada histology, serum NSE and urine VMA and confirmed type of health care system being a significant prognostic factor to predict treatment response after induction therapy with a p-value of 0.041 **(**Table 5**)**.

**Table 4:** Prognostic factors to predict treatment response after topotecan and cyclophosphamide containing induction therapy (univariate analysis)

	*Response ≥ 90%*	*Response < 90%*	*Crude OR* *(95% CI)*	*p-value*
*Age*	3.5 ± 2.5	4.1 ± 2.6	1.09 (0.93–1.28)	0.307
*Gender*				
*Male*	28 (45.2)	34 (54.8)	1	
*Female*	14 (31.1)	31 (68.9)	1.82 (0.82–4.08)	0.140
*Grade of differentiation*				
*Poorly differentiate & Undifferentiate*	26 (40.0)	39 (60.0)	1	
*Differentiate*	3 (37.5)	5 (62.5)	1.11 (0.24–5.06)	0.892
*MYNC*				
*Non-amplified*	17 (43.6)	22 (56.4)	1	
*Amplified*	3 (25.0)	9 (75.0)	2.32 (0.54–9.9)	0.256
*Shimada histology*				
*Favorable*	4 (44.4)	5 (55.6)	1	
*Unfavorable*	10 (43.5)	13 (56.5)	1.04 (0.22–4.91)	0.960
*NSE*	314.1 ± 398.9	415.7 ± 420.6	1.00 (1.00–1.00)	0.222
*VMA*	45.3 ± 93.3	22.7 ± 33.1	0.99 (0.99–1.00)	0.115
*Health care system*				
*University-based*	37 (45.7)	44 (54.3)	1	
*Community-based*	5 (19.2)	21 (80.8)	3.53 (1.21–10.28)	0.021

***Notes:*** *Data are categorical variables and presented as number (%) except for age, NSE and VMA which are continuous variables and presented as mean ± SD. Univariate analysis was calculated using logistic regression. p-value < 0.05 is considered as statistical significance*

***Abbreviations:*** *NSE* serum neuron-specific enolase (ng/mL); *VMA* urine vanillylmandelic acid (mg/day)

**Table 5:** Types of treatment centers associated with treatment response after topotecan and cyclophosphamide containing induction therapy (multivariate analysis)

*Health care system*	*Response ≥ 90%*	*Response < 90%*	*Crude OR* *(95% CI)*	*p-value*	*Adjusted OR* *(95% CI)*	*p-value*
*University-based*	37 (45.7)	44 (54.3)	1		1	
*Community-based*	5 (19.2)	21 (80.8)	3.53 (1.21–10.28)	0.021	3.13 (1.05–9.37)	0.041

***Notes:*** *Data are categorical variables and presented as number (%). Multivariate analysis was calculated using multiple logistic regression adjusted for age, gender, grade of tumor differentiation, MYCN status, Shimada histology, serum NSE and urine VMA. p-value < 0.05 is considered as statistical significance*

### Treatment-related toxicities after each topotecan containing induction cycle

Topotecan containing induction cycles (first and second induction cycles) were well tolerated with approximately 1 week required for hospitalization in which one third of the patients were re-admitted and less than 3% of those attended an intensive care unit. Time to neutrophil recovery was approximately 2 weeks duration. Common treatment-related toxicities included febrile neutropenia, mucositis and diarrhea in which the incidence was decreased at the second topotecan containing induction cycle compared with the first cycle. Rare treatment-associated adverse effects including typhlitis, renal dysfunction and elevated liver enzymes were reported at less than 1% among those high risk patients **(**Table 6**)**.

**Table 6:** Treatment-related toxicities after each cycle of topotecan and cyclophosphamide containing induction therapy

	*Data*	*First cycle*	*Second cycle*
*Hospital stay (days)*	Median (range)	7 (5–10)	5 (5–40)
*Nadir period (days)*	Median (range)	15 (10–20)	15 (10–20)
*Readmission after chemotherapy*	No	71 (66.4)	88 (82.2)
	Ward	33 (30.8)	18 (16.8)
	ICU	3 (2.8)	1 (0.9)
*Febrile neutropenia*	No	63 (58.9)	86 (80.4)
	Yes	44 (41.1)	21 (19.6)
*Mucositis*	No	84 (78.5)	93 (86.9)
	Yes	23 (21.5)	14 (13.1)
*Diarrhea*	No	102 (95.3)	103 (96.3)
	Yes	5 (4.7)	4 (3.7)
*Typhlitis*	No	106 (99.1)	106 (99.1)
	Yes	1 (0.9)	1 (0.9)
*Renal dysfunction*	No	106 (99.1)	106 (99.1)
	Yes	1 (0.9)	1 (0.9)
*Elevated liver enzymes*	No	105 (98.1)	106 (99.1)
	Yes	2 (1.9)	1 (0.9)

***Notes:*** *Data are presented as number (%) for categorical variables*

**Fig. 2:**
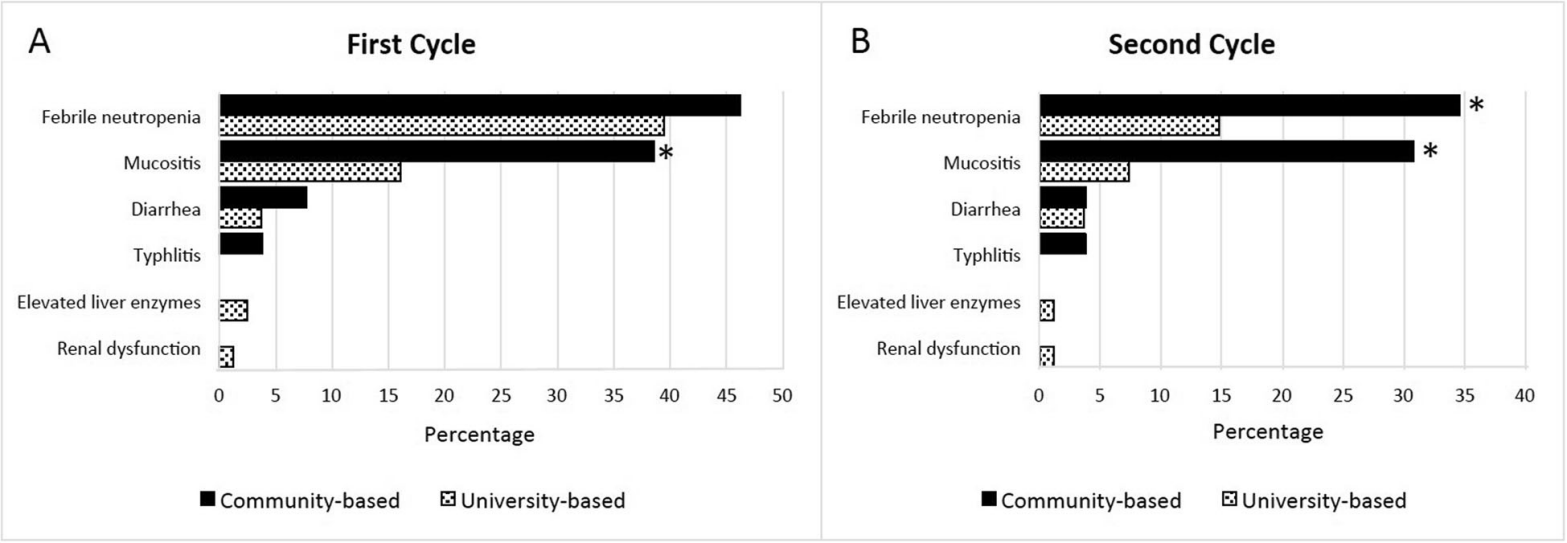
Treatment-related toxicities according to health care systems (university- versus community-based health care system) after first cycle (**a**) and second cycle (**b**) of topotecan and cyclophosphamide containing induction therapy . ***Notes:*** Graphs are presented as number (%). p-value was obtained from Fisher’s exact test, and p < 0.05 is statistically significant (*)

Moreover, treatment-related toxicities were also analyzed according to type of health care system (university-based versus community-based health care system) and were more significant in a community-based health care system compared with a university-based health care system. Interestingly, mucositis was significantly higher among patients treated at a community-based health care system in both cycles of topotecan and cyclophosphamide containing induction chemotherapy compared with those treated at a university-based health care system with a *p*-value of 0.016 and 0.005 in the first and second induction cycles, respectively. In addition, febrile neutropenia was significantly higher among patients undergone treatment at a community-based health care system in the second induction cycle compared with those treated at a university-based health care system with a p-value of 0.027 **(**Fig. 2**)**.

## Discussion

Unlike other childhood cancers, neuroblastoma carries unique characteristics including diverse clinical presentations depending on tumor location and various clinical outcomes based on tumor biology and disease stages [25]. For this reason, pre-treatment risk-stratification of patients with de-novo neuroblastoma are essential for treating physicians to implement an appropriate treatment plan for each individual. The INRG (International Neuroblastoma Risk Group) classification system has been widely used and based on patients’ age, disease stage at diagnosis, tumor histology, DNA ploidy, MYCN (n-myc) status and chromosome changes. Patients classified as low and intermediate risk groups typically have an excellent response to most treatment protocols [2, 4]. Unfortunately, patients with high risk neuroblastoma carry an extremely poor prognosis with dismal outcomes despite receiving intensive treatment. Additional innovative treatment such as immune or cellular therapy including chimeric antigen receptor (CAR) T-cell therapy, natural killer (NK) cell therapy or cytokine-induced killer (CIK) cell therapy, have been extensively studied with promising results [9, 26–28]. However, in developing countries with limited resources and where those additional innovative treatments are unavailable, modifying traditional intensive treatment might be the only way to improve patient outcomes. Herein, we incorporated topotecan, a cytotoxic agent extensively studied in relapsed and refractory solid tumors including neuroblastoma, in our induction regimen. In all, 107 patients with high risk neuroblastoma from various institutions in Thailand including university-based and community-based health care systems were enrolled in the study and all received topotecan and cyclophosphamide containing induction regimen during the first two induction cycles. After completing the 5th induction cycle, induction response was determined. Although MIBG and PET/CT scans are highly effective modalities to assess treatment response of primary tumor as well as metastatic soft tissues and bone sites [29], both functional imaging studies are not financially accessible for some patients and not available in some institutions. Therefore, bone scintigraphy was mainly used to assess bone involvement in high risk neuroblastoma patients enrolled in this study. Given the limited access to MIBG and PET/CT scans, the original INRC criteria of Brodeur et al., 1993 was used to assess treatment response [30]. Interestingly, all patients responded to treatment and nearly one half of those successfully achieved excellent treatment responses (CR and VGPR). According to the study from Matthay KK et al., patients with high risk neuroblastoma, achieving CR or VGPR post induction treatment, had significantly better survival rates than those who achieved lower responses even in the setting of chemotherapy alone with no hematopoietic stem cell transplantation [11]. These findings applied well to the current situation in our country and other developing countries where advanced consolidation treatments such as hematopoietic stem cell transplantation and immunotherapy are difficultly accessible. In addition, neuroblastoma was differentiated to a more maturing component described as ganglioneuroma in 6 patients. Those patients were initially diagnosed with high risk neuroblastoma from evidence of bone marrow involvement and elevated urine catecholamine metabolites, and had tissue pathologic specimen obtained after the fifth cycle of chemotherapy prior to surgical resection of the tumor according to protocol.

Moreover, we also investigated other potential prognostic factors that could be used to predict patient responses during induction treatment. Surprisingly, all well-known associated factors for high risk neuroblastoma including patient’s age, grade of tumor differentiation, MYCN status and Shimada histology did not significantly affect induction response.

These insignificant results could be from small sample sizes, and further study with a larger sample numbers might be needed. Moreover, if the patients have an evidence of metastasis at the time of diagnosis, they will automatically be stratified as high risk patients regardless of MYCN status. In addition, most neuroblastoma patients typically have metastatic disease at the time of diagnosis; therefore, MYCN tests were not then performed on those patients. Interestingly, the only important factor that could have had significant impact on induction response was the type of health care system. We found that high risk patients treated in a university-based health care system successfully achieved a better treatment response than those treated in a community-based health care system using both univariate and multivariate analyses. This finding might be because a university-based health care system has a higher capability to handle such complicated and difficult cases like high risk neuroblastoma. We have looked over the total dose, the timing of chemotherapy, duration of induction phase and the use of filgrastim and found no significant differences between two groups.

In addition to proving the effectiveness of topotecan and cyclophosphamide containing induction regimen to introduce satisfactory responses among patients with high risk neuroblastoma, most patients participating in this study tolerated the treatment well with no toxic death reported during induction therapy. Common treatment-associated adverse effects included febrile neutropenia, mucositis and diarrhea. However, those adverse effects were significantly more evident among patients treated in a community-based health care system than those treated in a university-based health care system. This might be the reason that type of health care system is a significant prognostic factor regarding induction response. The higher incidence of treatment-related toxicities among patients treated in a community-based health care system might affect the treatment schedule, e.g., the schedule might be delayed and those patients might not receive the next cycle of chemotherapy on time and subsequently be unable to achieve an optimal treatment response. This finding assures the importance of efficient supportive care in optimizing treatment outcomes of patients with high risk neuroblastoma. Early assessment of treatment-related adverse effects, proper supportive care measures and effective management of those complications are pivotal keys to reduce the occurrence of adverse effects and later results in minimizing modifications or treatment cancellations [31–34]. Moreover, several studies emphasized the importance of cancer treatment site which could significantly affect clinical outcomes of patients in which oncology patients treated at specialized comprehensive cancer centers had better survival compared with those treated at non-specialized comprehensive cancer facilities. Key sociodemographic factors were considered as barriers to care at specialized comprehensive cancer centers including patients’ ethnicity, insurance status, socioeconomic status and distance to treatment center [35–38].

The study’s limitation included the limited accessibility to highly effective functional imaging studies such as MIBG and PET/CT scans resulting in under estimated osteomedullary involvement which could affect the assessment of treatment response, and this might be the reason for a high number of patients who achieved CR/VGPR in this study comparing to those from other studies. In addition, a small sample size of this study might be another possibility for the differences in CR/VGPR patient’s numbers between our study and others. Although patients’ treatment response was meticulously determined by senior treating physicians and imaging studies were evaluated by experienced and skillful radiologists from each institution, the lack of central review was another limitation of our study which might affect an overall quality control measurement. The difficulty in interpreting bone scintigraphy might also account for the difference in VGPR vs PR rate in the two types of health care systems. In addition, the insignificant results of well-known prognostic factors for high risk neuroblastoma to predict induction response such as grade of tumor differentiation, MYCN status and Shimada histology could be from small sample sizes, and further study with a larger sample numbers might be needed. Since most neuroblastoma patients had metastatic disease at the time of diagnosis and were automatically stratified as high risk patients regardless of MYCN status, MYCN tests were not then performed on those patients.

## Conclusion

Although the use of topotecan and cyclophosphamide as an upfront chemotherapy for newly diagnosed high-risk neuroblastoma patients is new, our study affirms the clinical usefulness and safety of this combination in which the topotecan and cyclophosphamide containing induction regimen was effective against high risk neuroblastoma and able to provide optimal treatment response before consolidation treatment. The regimen is well tolerated with no toxic death and minimal adverse effects; therefore, it could be easily applied to institutions with limited resources in most developing countries. The most important predictive factor for induction response was the type of health care system, which might be related to the incidence of treatment-related adverse effects. Further study is required to determine long-term outcomes and survival rates of those patients with high risk neuroblastoma after completing consolidation treatment. In addition, the key differences between university- and community-based health care systems should be explored to narrow this gap and subsequently improve outcomes among all patients with high risk neuroblastoma.

## Data Availability

The datasets generated and/or analyzed during the current study are not publicly available since these datasets are being used in a different ongoing study; however, the datasets are available from the corresponding author on reasonable request.

## Abbreviations

ALT: Alanine aminotransferase
ANC: Absolute neutrophil count
Anti-GD2: hu14.18K322A or anti-disialoganglioside
AST: Aspartate aminotransferase
CAR: Chimeric antigen receptor
CIK: Cytokine-induced killer
CR: Complete response
DNA: Deoxyribonucleic acid
INRC: International neuroblastoma response criteria
INRG: International neuroblastoma risk group
INRGSS: International neuroblastoma risk group staging system
MIBG: Metaiodobenzylguanidine
MR: Mixed response
MYCN: N-myc or v-myc myelocytomatosis viral related oncogene, neuroblastoma derived (avian)
NK cell: Natural killer cell
NR: No response
NSE: Neuron-specific enolase
PD: Progressive disease
PR: Partial response
SD: Standard deviation
SPSS: Statistical package for the social science
ThaiPOG: Thai pediatric oncology group
ThaiPOG-NB-13HR: Thai pediatric oncology group protocol for high risk neuroblastoma
VGPR: Very good partial response
VMA: Vanillylmandelic acid
